# Distinct and shared neuropsychiatric phenotypes in FTLD-tauopathies

**DOI:** 10.3389/fnagi.2023.1164581

**Published:** 2023-06-09

**Authors:** Rachel Keszycki, Allegra Kawles, Grace Minogue, Antonia Zouridakis, Alyssa Macomber, Nathan Gill, My Vu, Hui Zhang, Christina Coventry, Emily Rogalski, Sandra Weintraub, M-Marsel Mesulam, Changiz Geula, Tamar Gefen

**Affiliations:** ^1^Mesulam Center for Cognitive Neurology and Alzheimer’s Disease, Feinberg School of Medicine, Northwestern University, Chicago, IL, United States; ^2^Department of Psychiatry and Behavioral Sciences, Feinberg School of Medicine, Northwestern University, Chicago, IL, United States; ^3^Department of Preventative Medicine, Feinberg School of Medicine, Northwestern University, Chicago, IL, United States; ^4^Department of Cell and Molecular Biology, Feinberg School of Medicine, Northwestern University, Chicago, IL, United States

**Keywords:** neuropsychiatric symptoms, frontotemporal lobar degeneration, primary progressive aphasia, behavioral variant frontotemporal dementia, pick disease, corticobasal degeneration, progressive supranuclear palsy

## Abstract

Frontotemporal lobar degeneration (FTLD) with tau pathology (FTLD-tau) commonly causes dementia syndromes that include primary progressive aphasia (PPA) and behavioral variant frontotemporal dementia (bvFTD). Cognitive decline in PPA and bvFTD is often accompanied by debilitating neuropsychiatric symptoms. In 44 participants with PPA or bvFTD due to autopsy-confirmed FTLD-tau, we characterized neuropsychiatric symptoms at early and late disease stages and determined whether the presence of certain symptoms predicted a specific underlying FTLD-tauopathy. Participants completed annual research visits at the Northwestern University Alzheimer’s Disease Research Center. All participants had an initial Global Clinical Dementia Rating (CDR) Scale score ≤ 2, and neuropsychiatric symptoms were evaluated *via* the Neuropsychiatric Inventory-Questionnaire (NPI-Q). We assessed the frequency of neuropsychiatric symptoms across all participants at their initial and final visits and performed logistic regression to determine whether symptoms predicted a specific FTLD-tau pathologic diagnosis. Across the FTLD-tau cohort, irritability and apathy were most frequently endorsed at initial and final visits, respectively, whereas psychosis was highly uncommon at both timepoints. Irritability at initial visit predicted greater odds of a 4-repeat compared to a 3-repeat tauopathy (OR = 3.95, 95% CI = 1.10–15.83, *p* < 0.05). Initial sleep disturbance predicted greater odds of progressive supranuclear palsy (PSP) compared to other FTLD-tau subtypes (OR = 10.68, 95% CI = 2.05–72.40, *p* < 0.01). Appetite disturbance at final evaluation predicted lower odds of PSP (OR = 0.15, 95% CI = 0.02–0.74, *p* < 0.05). Our findings suggest that characterization of neuropsychiatric symptoms can aid in the prediction of underlying FTLD-tauopathies. Given considerable pathologic heterogeneity underlying dementias, neuropsychiatric symptoms may be useful for differential diagnosis and treatment planning.

## 1. Introduction

Frontotemporal lobar degeneration (FTLD) with tau pathology refers to a group of neurodegenerative diseases characterized by pathological accumulation of 3-repeat (3R) or 4-repeat (4R) tau species (Dickson et al., 2011; Irwin et al., 2015). The 3R FTLD-tau subtype is Pick disease (PiD), whereas the 4R subtypes include corticobasal degeneration (CBD) and progressive supranuclear palsy (PSP). FTLD-tauopathies typically cause clinical dementia syndromes with symptom onset before age 65 (Irwin et al., 2015). The most common clinical phenotypes include primary progressive aphasia (PPA), characterized by initially isolated language decline (Mesulam et al., 2021), and behavioral variant frontotemporal dementia (bvFTD), characterized by changes in comportment and personality with or without executive dysfunction (Rascovsky et al., 2011). Heterogeneity in the underlying neuropathologies causing PPA and bvFTD presents a challenge to differential diagnosis and treatment, and clinicians often employ a combination of antemortem biomarkers. However, FTLD-tau is diagnosed at autopsy, and there are currently no definitive, antemortem biomarkers to diagnose FTLD-tauopathies. Outside of the “primary” neuropathologic etiology driving clinical symptoms, most patients with neurodegenerative dementias also have one or more comorbid neuropathologies, such as Alzheimer’s disease neuropathologic change (ADNC), that may impact clinical phenotype and disease trajectory and further complicate differential diagnosis and treatment (Spires-Jones et al., 2017).

Over the course of the disease, deficits in PPA and bvFTD progress to impact patients’ functional status and are often coupled with devastating neuropsychiatric symptoms (Banks and Weintraub, 2008; Scarioni et al., 2020). In addition to pathological heterogeneity, the presence of neuropsychiatric symptoms in this younger patient population, particularly early in the disease course, may obfuscate differential diagnosis from primary psychiatric disorders (Ducharme et al., 2020). Neuroimaging studies suggest considerable overlap in frontal, limbic, and subcortical circuits and in monoaminergic brainstem nuclei that are vulnerable to FTLD-tau pathologies and are implicated in both primary psychiatric disorders and neuropsychiatric symptoms (Peet et al., 2021; Scarioni et al., 2023). Accordingly, neuropsychiatric symptoms in neurodegenerative dementias are often treated with existing psychiatric medications (Le and Finger, 2021); however, these approaches are non-standardized, and there are currently no FDA-approved medications for this purpose.

The present study investigated the following in a cohort of participants with PPA and bvFTD due to autopsy-confirmed FTLD-tau: (1) the distinct and shared neuropsychiatric phenotypes of FTLD-tauopathies and (2) whether the presence of certain neuropsychiatric symptoms predicts the odds of specific underlying FTLD-tauopathies. Characterization of neuropsychiatric symptoms in FTLD-associated dementias has significant implications for prognosis, diagnosis, and refinement of antemortem biomarkers for underlying pathology, particularly in early stages of disease when symptoms are most distinct and responsive to intervention (Dickson et al., 2011; Irwin et al., 2015).

## 2. Methods

Participants with PPA or bvFTD completed two or more research visits at the Northwestern University Alzheimer’s Disease Research Center from 2006 to 2020. Participants underwent neuropsychological testing at each visit following the National Alzheimer’s Coordinating Center (NACC) Uniform Data Set (UDS) Versions 2.0 (Weintraub et al., 2009) and 3.0 (Weintraub et al., 2018), which occurred approximately annually (M = 1.19, SD = 0.63 years between visits). Most PPA participants were enrolled through Northwestern’s PPA Research Program. A consensus of clinicians rated dementia severity at each visit *via* the Clinical Dementia Rating (CDR) Scale (Morris, 1993). Study partners, most of whom were primary caregivers, completed the informant-based Neuropsychiatric Inventory-Questionnaire (NPI-Q) (Kaufer et al., 2000), answering whether participants exhibited 12 core neuropsychiatric symptoms in the month prior to their research visit. The following symptoms were assessed: apathy, disinhibition, motor stereotypies, appetite disturbance, depression, anxiety, elation, irritability, delusions, hallucinations, sleep disturbance, and agitation. This study focused analyses on neuropsychiatric symptoms from participants’ initial and final research visits to gauge neuropsychiatric phenotypes at earlier and later stages of disease, respectively. A Northwestern University institutional review board approved this study.

Participants consented to brain donation and underwent semiquantitative neuropathological evaluation at autopsy for gross atrophy, pathologic inclusions, neuronal loss and gliosis, and superficial microvacuolation (Gefen et al., 2020a). We initially identified 60 cases with PPA or bvFTD due to primary PiD, PSP, or CBD neuropathology using established clinical (Rascovsky et al., 2011; Mesulam et al., 2021) and neuropathologic (Dickson et al., 2011) criteria. We then excluded cases with an initial Global CDR score > 2 (i.e., “severe” dementia) and/or those with NPI-Q data for only one research visit. Cases with comorbid “high” Alzheimer’s disease neuropathological change (ADNC), “severe” cerebrovascular disease, and/or neocortical or limbic Lewy body disease were also excluded. Our final sample consisted of 44 participants (50% female) with PPA (*N* = 21) or bvFTD (*N* = 23) due to FTLD-tau who met these criteria (see Table 1).

**Table 1:** Participant characteristics and demographics.

	PiD (*n* = 17)	PSP (*n* = 10)	CBD (*n* = 17)	Total (*n* = 44)
**Gender – *n* (%)**				
Male	9 (52.94%)	5 (50%)	8 (47.06%)	22 (50%)
Female	8 (47.06%)	5 (50%)	9 (52.94%)	22 (50%)
**Education (years)**				
M (SD)	15.76 (2.91)	16.30 (1.89)	15.76 (1.99)	15.89 (2.32)
APOE Allele* – *n* (%)				
ε2	2 (7.14%)	2 (10%)	1 (3.85%)	5 (6.76%)
ε3	22 (78.57%)	17 (85%)	20 (76.92%)	59 (79.73%)
ε4	4 (14.29%)	1 (5%)	5 (19.23%)	10 (13.51%)
**Comorbid ADNC – *n* (%)**				
Yes	9 (52.94%)	5 (50%)	9 (52.94%)	23 (52.27%)
No	8 (47.06%)	5 (50%)	8 (47.06%)	21 (47.73%)
Initial CDR scores* – M (SD)				
Standard Global	0.85 (0.58)	0.55 (0.50)	0.47 (0.51)	0.64 (0.55)
Supplemental	n = 13	n = 7	n = 15	n = 35
Comportment	1.19 (0.93)	0.86 (0.90)	0.73 (0.84)	0.93 (0.88)
Language	1.00 (0.71)	1.29 (1.22)	1.07 (0.78)	1.09 (0.84)
**Age at onset (years)**				
M (SD)	59.06 (6.49)^b^	71 (8.03)	62.71 (5.83)^a^	63.18 (7.93)
**Age at death (years)**				
M (SD)	69.18 (6.04)^c^	80.40 (8.63)	71 (8.30)^b^	72.43 (8.09)
**Disease duration (years)**				
M (SD)	10.12 (2.98)	9.30 (5.14)	8.29 (3.79)	9.23 (3.85)

Significant difference compared to PSP at ^a^*p* < 0.05, ^b^*p* < 0.01, and ^c^*p* < 0.001. PiD, Pick disease; CBD, corticobasal degeneration; PSP, progressive supranuclear palsy; ADNC, Alzheimer’s disease neuropathologic change; CDR, Clinical Dementia Rating Scale. *APOE allele status was unavailable for *N* = 3 PiD and *N* = 4 CBD participants. Initial supplemental CDR scores unavailable for *N* = 4 PiD, *N* = 3 PSP, and *N* = 2 CBD participants.

Statistical analyses were performed in RStudio (v1.1.456). We conducted one-way Welch’s ANOVA with Tukey post-hoc tests to compare FTLD-tau subtype group demographics. We performed Fisher’s exact test to assess whether the frequency of APOE alleles or comorbid ADNC pathology differed by FTLD-tau subtype group. We conducted logistic regression to determine whether specific NPI-Q symptoms predicted FTLD-tau species (3R or 4R) or subtype (PiD, CBD, or PSP) at autopsy. Regressions covaried for the proportion of each participant’s total disease duration elapsed [i.e., (disease duration at visit_i_/total disease duration_i_)].

## 3. Results

### 3.1. Participant characteristics and demographics

The PSP group was significantly older at symptom onset (*F*_2, 21.81_ = 7.83, *p* < 0.01) than the PiD (*p* < 0.01) and CBD (*p* < 0.05) groups. Accordingly, age at death was also significantly older for PSP participants (*F*_2, 24.52_ = 11.68, *p* < 0.001) compared to PiD (*p* < 0.001) and CBD (*p* < 0.01) participants. Education, initial Global CDR score, and disease duration did not significantly differ between FTLD-tau subtype groups. Among 37 participants with APOE genotype available, 80% of alleles were ε3, and the distribution of APOE alleles did not differ significantly by FTLD-tau subtype. Secondary ADNC (mild or intermediate only) was present in 52.3% of total FTLD-tau cases at autopsy, and the frequency of comorbid ADNC did not vary significantly between FTLD-tau groups. Final visits occurred within a year before death on average (M = 0.71; SD = 0.66 years). Refer to Table 1 for participant characteristics and demographic information.

### 3.2. Frequency of neuropsychiatric symptoms across all FTLD-tau participants

**Figure 1:**
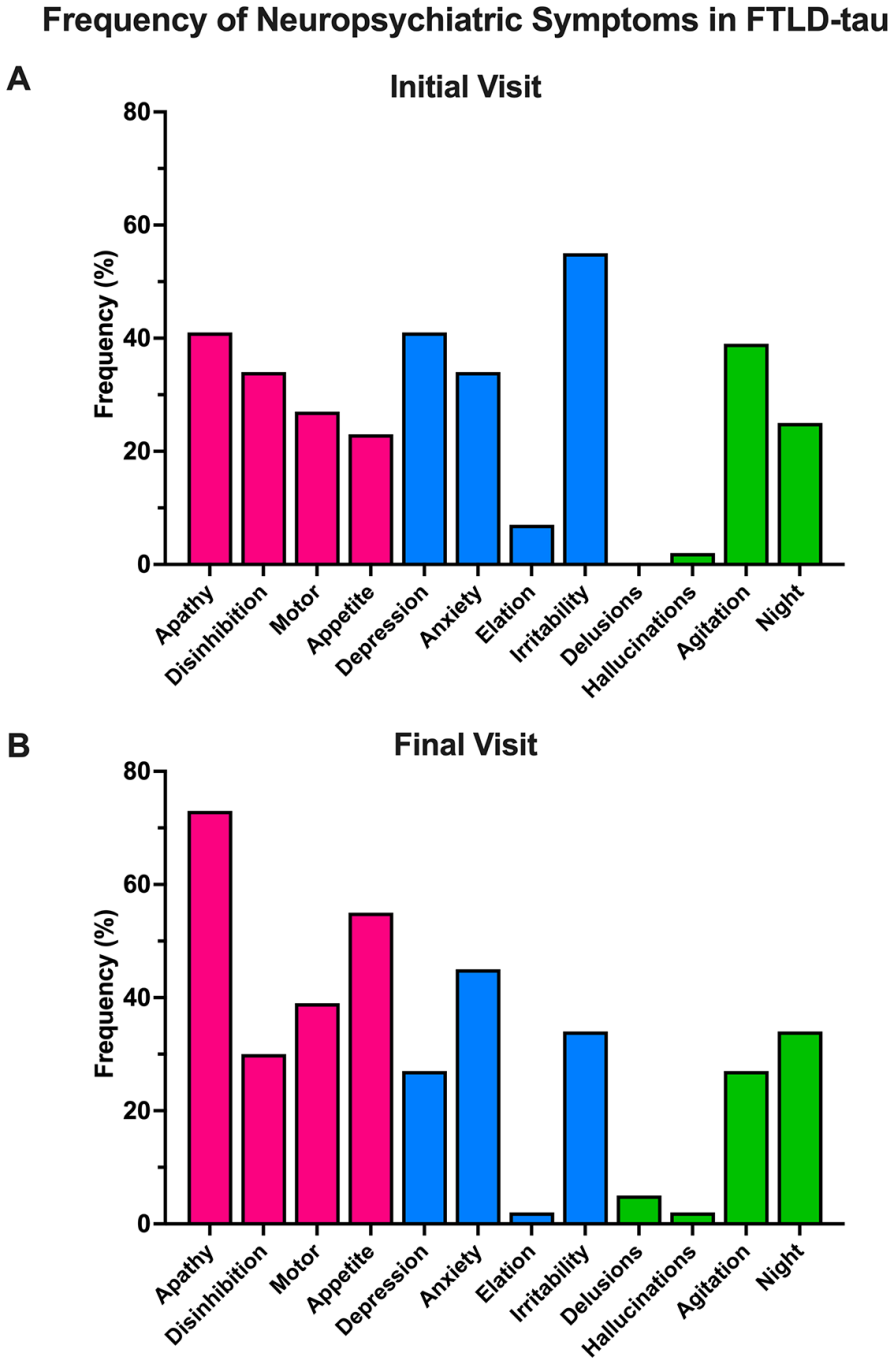
Frequency of neuropsychiatric symptoms in FTLD-tau. **(A)** Across the cohort, symptoms of behavioral/comportmental disruption (pink) and affective disturbance (blue) were common at initial visit. These categories are based on Banks and Weintraub (2008). Irritability was the most frequently endorsed symptom initially in 55% of the cohort. Psychotic symptoms (i.e., delusions and hallucinations) were infrequently endorsed (0–2%). **(B)** At final visit, symptoms of behavioral/comportmental disruption became more prominent. Apathy was the most frequently endorsed symptom at this timepoint (73%) and was followed by appetite disturbance (55%). Psychosis was again uncommon (2–5%).

Irritability was the most frequently endorsed symptom across the cohort at initial visit (55% endorsed) followed by apathy and depression (41% endorsed for both) (Figure 1A). At initial visit, 54.5% of participants were prescribed psychoactive medications based on report collected by the NACC UDS; of these medications, 35.3% were acetylcholinesterase or cholinesterase inhibitors (e.g., memantine), and 32.4% were selective serotonin reuptake inhibitors (SSRIs, e.g., escitalopram) (Supplementary Figure 1A). At final visit, the most endorsed symptom across all participants was apathy (73% endorsed) followed by appetite disturbance (55% endorsed) (Figure 1B). The frequency of participants prescribed psychoactive medication increased to 77.3%, and SSRIs were most common, comprising 30.8% of these prescriptions (Supplementary Figure 1B). Psychosis (i.e., delusions and hallucinations) was highly uncommon at both timepoints (0–5% endorsed).

### 3.3. Prediction of FTLD-tau pathologic diagnosis given neuropsychiatric symptom endorsement

#### 3.3.1. FTLD-tau species (3R or 4R)

**Figure 2:**
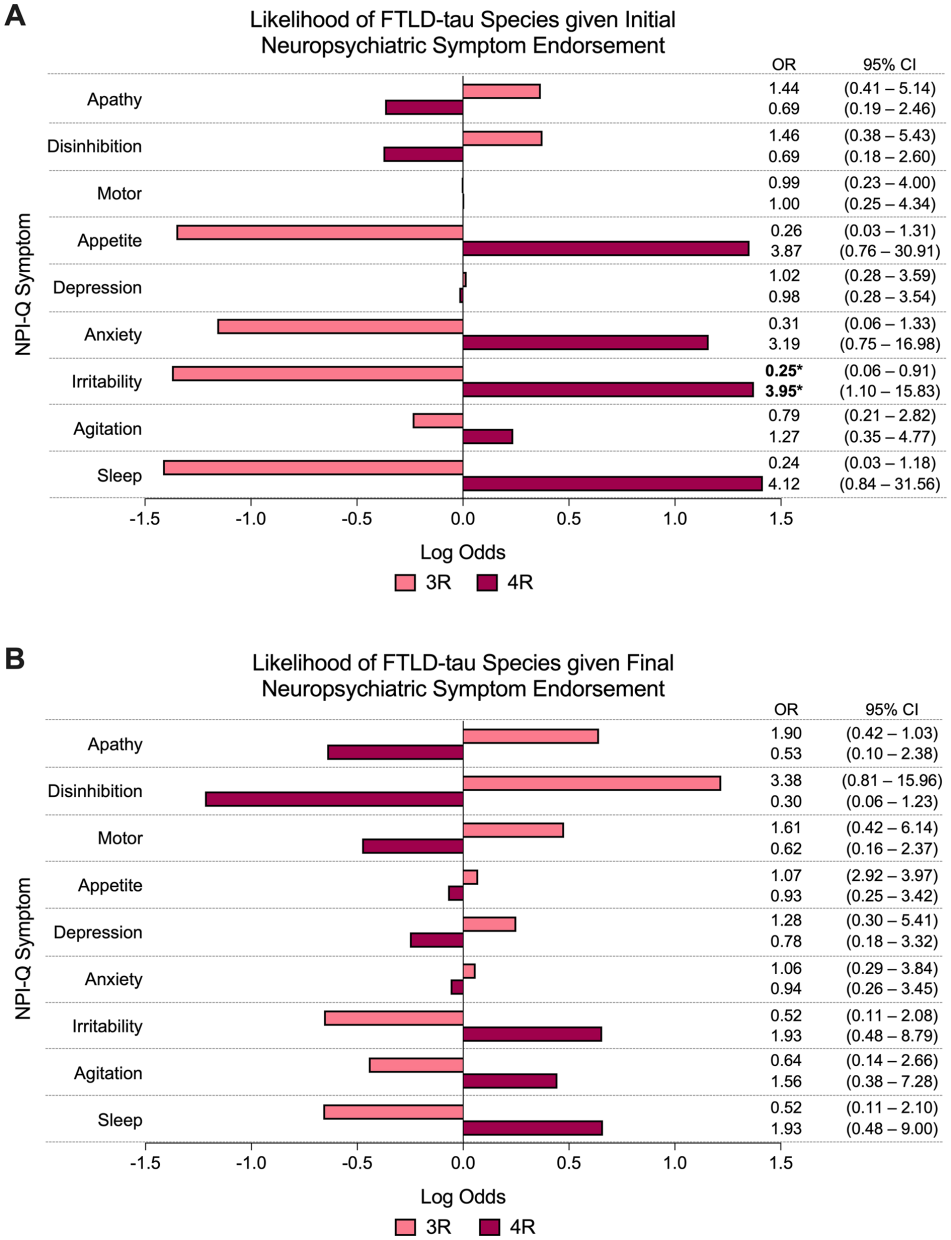
Odds of FTLD-tau species given neuropsychiatric symptom endorsement. **(A)** At initial visit, the odds of having a 4R FTLD-tauopathy at autopsy was significantly greater than the odds of having a 3R FTLD-tauopathy if irritability was endorsed. Other NPI-Q symptoms did not reliably predict FTLD-tau species at **(A)** initial or **(B)** final visit. Bars depict the log odds determined *via* logistic regressions controlling for disease duration at time of visit. Exponentiated odds ratios and 95% confidence intervals are presented to the right of the graph. Sample sizes are *N* = 27 4R FTLD-tau and *N* = 17 3R FTLD-tau. *p < 0.05.

At initial visit, the presence of irritability predicted significantly greater odds of having a 4R FTLD-tauopathy (i.e., CBD or PSP) at autopsy compared to a 3R (PiD) FTLD-tauopathy (67% 4R vs. 35% 3R endorsed, OR = 3.95, 95% CI = 1.10–15.83, *p* < 0.05; Figure 2A). Endorsement of other NPI-Q symptoms at initial visit did not reliably predict FTLD-tau species. NPI-Q symptoms were not significant predictors of FTLD-tau species at final visit (Figure 2B).

#### 3.3.2. FTLD-tau subtype (PSP, CBD, or PiD)

**Figure 3:**
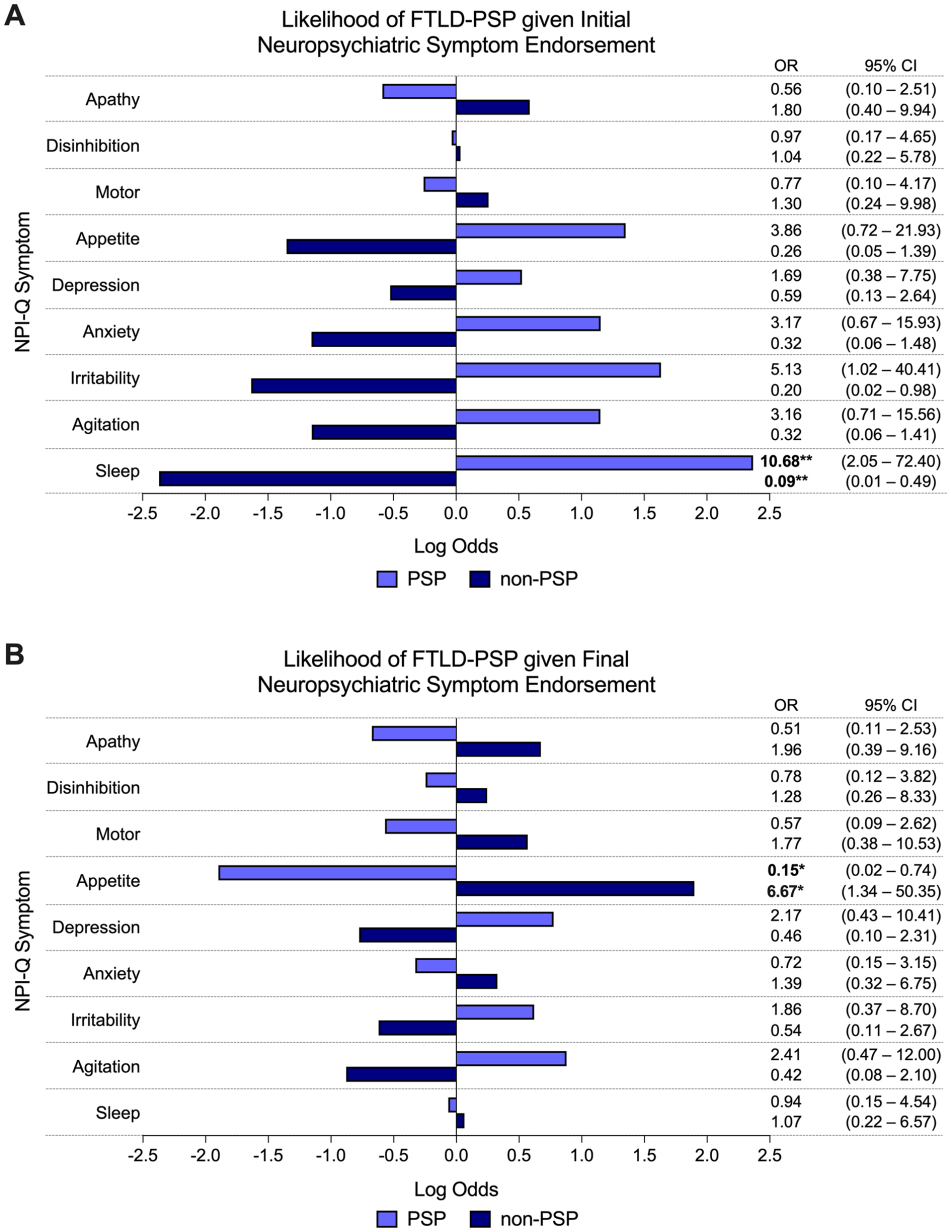
Odds of FTLD-PSP given neuropsychiatric symptom endorsement. **(A)** The odds of PSP pathologic diagnosis were significantly greater than other FTLD-tau subtypes (i.e., PiD and CBD) if sleep disturbance was endorsed at initial visit. Other individual NPI-Q symptoms did not reliably predict pathologic diagnosis of PSP (or other FTLD-tau subtype) at initial endorsement. **(B)** Endorsement of appetite disturbance at final visit predicted significantly lower odds of PSP pathologic diagnosis compared to non-PSP subtypes. Final endorsement of other neuropsychiatric symptoms did not reliably predict underlying PSP (or other FTLD-tau subtypes). Bars depict the log odds determined *via* logistic regressions controlling for disease duration at time of visit. Exponentiated odds ratios and 95% confidence intervals are presented to the right of the graph. Sample sizes are *N* = 10 FTLD-PSP and *N* = 34 FTLD-CBD or FTLD-PiD. *p < 0.05, **p < 0.01.

The presence of sleep disturbance at initial visit predicted significantly greater odds of PSP pathologic diagnosis compared to non-PSP (i.e., PiD and CBD) subtypes (60% PSP vs. 15% non-PSP endorsed, OR = 10.68, 95% CI = 2.05–72.40, *p* < 0.01; Figure 3A). Other NPI-Q symptoms did not reliably predict FTLD-tau subtype at initial visit. At final visit, the presence of appetite disturbance predicted significantly lower odds of PSP pathologic diagnosis compared to non-PSP subtypes (20% PSP vs. 65% non-PSP endorsed, OR = 0.15, 95% CI = 0.02–0.74, *p* < 0.05; Figure 3B). Endorsement of other neuropsychiatric symptoms was not a significant predictor of FTLD-tau subtype at final visit.

## 4. Discussion

To our knowledge, this is the first study to characterize neuropsychiatric phenotypes in a longitudinally evaluated case series of patients with PPA and bvFTD due to autopsy-confirmed FTLD-tau. Analyses led to 3 main findings: (1) patients with PPA or bvFTD due to FTLD-tau commonly present with symptoms of affective disturbance (e.g., irritability, depression) and behavioral/comportmental disruption (e.g., apathy, appetite disturbance) but not psychosis, (2) the presence of initial irritability predicts greater odds of having an underlying 4R FTLD-tauopathy, and (3) the presence of early-stage sleep disturbance and late-stage appetite disturbance predict greater and lesser odds, respectively, of PSP pathologic diagnosis.

As with prior studies, symptoms of affective and behavioral/comportmental disruption were common in the mild to moderate stages of FTLD-tau overall with apathy predominating in later stages (Banks and Weintraub, 2008; Castillo-García et al., 2022; see Figure 1). Altered amygdala activation and connectivity to the ventromedial prefrontal cortex (vmPFC), orbitofrontal cortex (OFC), and anterior cingulate cortex (ACC) are implicated in both primary mood disorders (Frodl et al., 2002; Makovac et al., 2016) and in affective neuropsychiatric symptoms in dementia (Poulin et al., 2011; Trnka et al., 2018). In FTLD, symptoms of behavioral/comportmental disruption, particularly apathy and/or disinhibition, are hypothesized to result from altered connectivity between the vmPFC, OFC, ACC, thalamus, and striatum (Massimo et al., 2009; Rohrer and Warren, 2010; Fernández-Matarrubia et al., 2018). The prominence of affective and behavioral/comportmental symptoms in our cohort map well onto studies of FTLD-tau showing that the aforementioned regions undergo significant atrophy and tau accumulation (Irwin et al., 2016; Kovacs et al., 2020).

Psychosis was nearly absent in our FTLD-tau cohort, paralleling prior reports of infrequent delusions and visual hallucinations in FTLD-tau compared to Lewy body disease or FTLD with TDP-43 pathology (Scarioni et al., 2020; Naasan et al., 2021). Importantly, while past studies incorporated heterogenous clinical and pathologic diagnoses, we report these findings in a cohort of participants diagnosed clinically with only PPA or bvFTD due to FTLD-tau and excluding significant comorbid pathologies. The exact neuroanatomic correlates of psychosis in neurodegenerative diseases are unknown; however, studies suggest that aberrant parieto-occipital connectivity may underlie visual hallucinations, whereas disruption to frontal regions and the hippocampus are implicated in delusions (Nagahama et al., 2010; Ismail et al., 2022; Scarioni et al., 2023). As differential diagnosis of FTLD-tau from Lewy body disease and FTLD with TDP-43 pathology may be complicated by overlapping clinical symptoms and neuroimaging biomarkers (Duignan et al., 2021), the absence of psychosis may represent a useful indicator of FTLD-tauopathies, potentially resolving some of this diagnostic ambiguity.

Within our FTLD-tau cohort, irritability early in the disease course predicted greater odds of developing a 4R versus a 3R FTLD-tauopathy. While studies of neuropsychiatric symptoms between tau species in PPA and bvFTD are limited, one group (Tighe et al., 2012) found that reduced white matter integrity of the anterior cingulum was associated with increased odds of irritability in mild cognitive impairment and dementia of the Alzheimer’s type. Given that 4R tauopathies present with more abundant glial pathology compared to 3R/PiD, degeneration of limbic white matter tracts may also contribute to irritability in mild-to-moderate PSP and CBD, although further research is needed.

It is debated whether PSP and CBD—both subtypes of 4R FTLD-tau species—are part of a pathologic spectrum or separate disease entities (Dickson et al., 2011). Findings of a recent study indicate distinct filament folds between PSP and CBD, which may impact patterns of temporospatial disease progression in the brain and their corresponding clinical phenotypes (Shi et al., 2021). In the present study, we found a distinct neuropsychiatric phenotype for PSP from that of PiD and CBD. In early-stage FTLD-tau, the presence of sleep disturbance predicted greater odds of PSP pathologic diagnosis. Studies of sleep disruption in PSP (Walsh et al., 2016, 2017) are limited to non-autopsied samples with additional clinical syndromes. Nevertheless, impaired circadian rhythms, sleep/wake cycles, and REM sleep in PSP have been reported (Walsh et al., 2016, 2017), potentially due to early basal forebrain degeneration and dysregulation of arousal (Dickson et al., 2011). Significant atrophy of brainstem structures is also hypothesized to cause dysphagia in PSP (Clark et al., 2020), which has been linked to reduced food intake and accelerated weight loss (Maetzler et al., 2016; Tsuge et al., 2016). We found that appetite disturbance predicted lower odds of PSP in late-stage FTLD-tau. Therefore, this finding may reflect impulsive eating behaviors, like binging or hyperorality, in PiD and CBD (Rascovsky et al., 2011). The etiology of these behaviors may involve tau accumulation in posterior hypothalamic nuclei, impairing inhibitory regulation of feeding pathways (Piguet et al., 2011). Frontal cortical atrophy, which is relatively greater in CBD and PiD compared to PSP, may lead to further disinhibition of these behaviors (Dickson et al., 2011; Irwin et al., 2015).

One limitation of the present study is its small sample size, which may limit generalizability. Additionally, although the NPI-Q is a well-validated and widely used measure of neuropsychiatric symptoms in dementia, it is brief and reliant on informant self-report. We previously reported that non-autopsied participants with PPA or bvFTD showed distinct phenotypes of clinical symptoms on the FTLD Module of the UDS, which is comprised of psychometric assessments and questionnaires designed to better capture impairments in FTLD specifically (Gefen et al., 2020b). A more nuanced investigation of neuropsychiatric symptoms in FTLD-tau utilizing the FTLD Module or additional measures of prominent symptoms in this population represents a promising direction for future research.

As the nosology of FTLD evolves, so too will biomarker development and intervention strategies. Our findings suggest that distinct neuropsychiatric symptoms may aid in characterizing and differentiating FTLD-tauopathies. These results highlight the utility of closely monitoring neuropsychiatric symptoms in FTLD to identify potential indicators of underlying pathology and to facilitate treatment planning.

## Data availability statement

The datasets presented in this article are not readily available because data will be made available to researchers whose proposed use of the data has been approved with a signed data access agreement. Requests to access the datasets should be directed to RK, RachelKeszycki2024@u.northwestern.edu.

## Ethics statement

The studies involving human participants were reviewed and approved by Northwestern University Institutional Review Board. The patients/participants provided their written informed consent to participate in this study.

## Author contributions

RK, AK, CC, ER, SW, M-MM, CG, and TG jointly designed the study. RK, AK, NG, GM, AZ, MV, HZ, CC, ER, SW, and M-MM assisted in data collection and interpretation. RK, NG, MV, and HZ contributed to statistical analysis and code writing. RK, AK, GM, AZ, AM, CG, and TG wrote and edited the manuscript. All authors contributed to the article and approved the submitted version.

## Funding

This work was supported by grants from the National Institute on Aging (P30AG072977, R01NS085770, R01NS075075, R01AG056258, R01AG062566, and R01AG077444), National Institute on Deafness and Other Communication Disorders (R01DC008552), and the National Alzheimer’s Coordinating Center (U01AG016976); by an institutional training grant from the National Institute of Neurological Disorders and Stroke (T32NS047987); by a training grant from the National Institute on Aging (F31AG07631); and by a training grant from the National Science Foundation (DGE1842165).

## Conflict of interest

The authors declare that the research was conducted in the absence of any commercial or financial relationships that could be construed as a potential conflict of interest.

## Publisher’s note

All claims expressed in this article are solely those of the authors and do not necessarily represent those of their affiliated organizations, or those of the publisher, the editors and the reviewers. Any product that may be evaluated in this article, or claim that may be made by its manufacturer, is not guaranteed or endorsed by the publisher.
