# Supplementary material for: Distinct and shared neuropsychiatric phenotypes in FTLD-tauopathies
Source: Front Aging Neurosci. 2023 Jun 9;15:1164581. doi: 10.3389/fnagi.2023.1164581 (PMC10289868; doi:10.3389/fnagi.2023.1164581)
Supplement: Supplementary file 1 [file Data_Sheet_1.PDF]

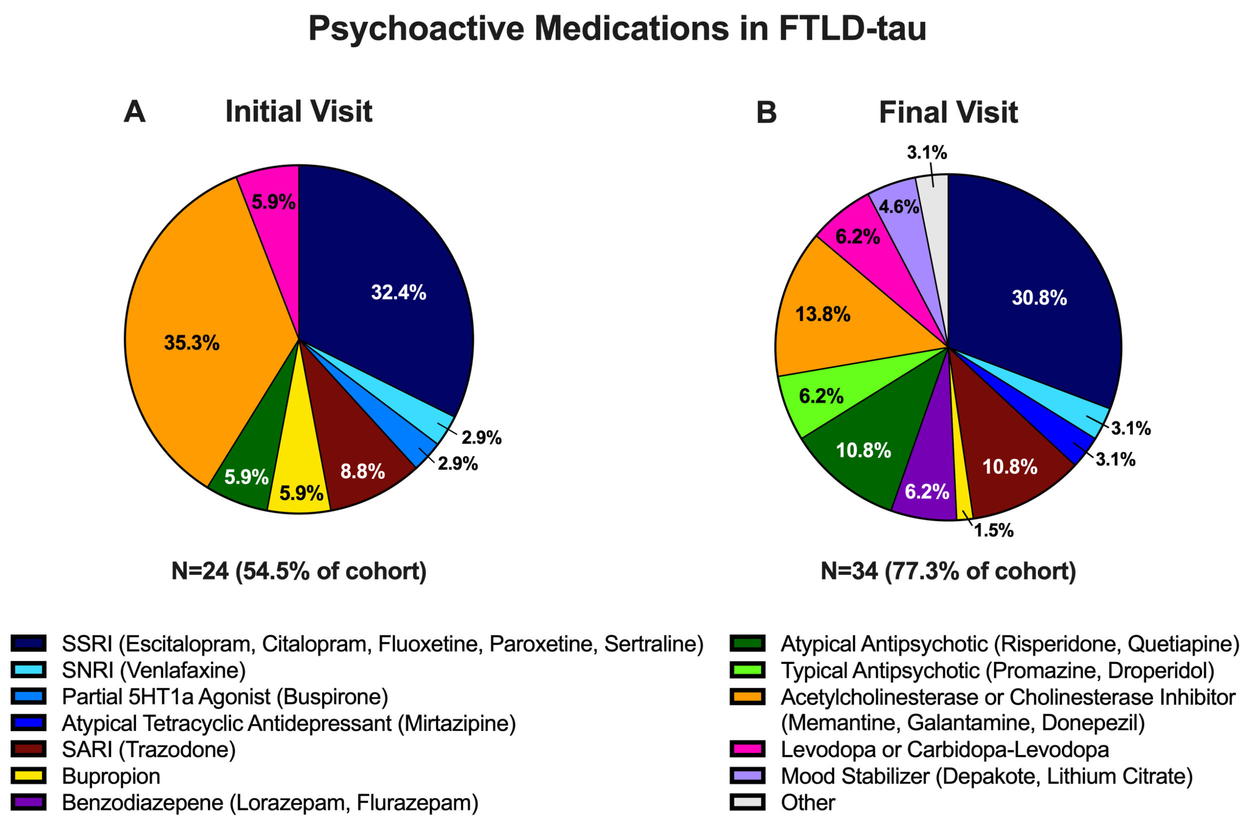

Supplementary Figure 1. Psychoactive Medications in FTLD-tau (reported via UDS Versions 2.0 and 3.0). A) At initial visit, 24 participants, or 54.5% of the total cohort, were prescribed psychoactive medications, most commonly acetylcholinesterase or cholinesterase inhibitors (35.3%) or selective serotonin reuptake inhibitors (SSRIs, 32.4%). **B)** The number of participants prescribed psychoactive medications increased to 34, or 77.3% of the total cohort, at final visit. Of the prescribed medications, SSRIs comprised 30.8% of prescribed medications at final visit. Note: SSRI, selective serotonin reuptake inhibitor; SNRI, serotonin and norepinephrine reuptake inhibitor; SARI, serotonin antagonist and reuptake inhibitor.
